# Supplementary material for: Living with Overweight, Rather than a History of Gestational Diabetes, Influences Dietary Quality and Physical Activity during Pregnancy
Source: Nutrients. 2022 Feb 3;14(3):651. doi: 10.3390/nu14030651 (PMC8837922; doi:10.3390/nu14030651)
Supplement: Supplementary file 1 [file nutrients-14-00651-s001.zip › nutrients-1581395-supplementary.pdf]

Table S1. Home region of the study participants and percentage of births in the area in 2017.

Area	Births in the area[a], %	Home region in the study population, %
Southern Finland	43.6	43.6
Western Finland	34.6	38.7
Eastern Finland	8.5	7.1
Oulu region	9.7	7.2
Lapland	2.9	3.4
Åland	0.5	0.1

[a]Calculated from the Finnish perinatal statistics 2017.

Table S2. The frequency of using dietary supplements in all the women, in the women with and without a history of gestational diabetes and in the women with normal weight and the women with overweight/obesity.

	All women	Women with no history of GDM	Women with a history of GDM	Women with normal weight	Women with over-weight/obesity		
	n = 1034	*n* = 383	*n* = 86	*n* = 656	*n* = 378		
	n (%)	*n* (%)	*n* (%)	*n* (%)	*n* (%)	*p*-value[a]	*p*-value[b]
Multivitamin						0.053[c]	0.003[c]
5 to 7 times per week	753 (76.8)	273 (75.8)	63 (76.8)	464 (74.8)	289 (80.1)		
1 to 4 times per week	46 (4.7)	19 (5.3)	8 (9.8)	23 (3.7)	23 (6.4)		
Less than once a week	10 (1.0)	2 (0.6)	1 (1.2)	8 (1.3)	2 (0.6)		
Does not use	171 (17.4)	66 (18.3)	9 (11.0)	125 (20.2)	46 (12.7)		
Vitamin D						0.61[c]	0.31[c]
5 to 7 times per week	367 (43.1)	131 (41.9)	27 (38.0)	251 (45.4)	116 (38.8)		
1 to 4 times per week	71 (8.3)	28 (8.9)	10 (14.1)	45 (8.1)	26 (8.7)		
Less than once a week	20 (2.3)	11 (3.5)	1 (1.4)	13 (2.4)	7 (2.3)		
Does not use	389 (45.7)	140 (44.7)	33 (46.5)	242 (43.8)	147 (49.2)		
Folic acid						0.70[c]	0.34[c]
5 to 7 times per week	344 (40.7)	111 (36.4)	21 (30.0)	222 (41.0)	122 (40.3)		
1 to 4 times per week	29 (3.4)	13 (4.3)	2 (2.9)	20 (3.7)	9 (3.0)		
Less than once a week	11 (1.3)	4 (1.3)	0 (0.0)	10 (1.8)	1 (0.3)		
Does not use	457 (54.1)	175 (57.4)	47 (67.1)	288 (53.1)	169 (55.8)		
Calcium						0.97[c]	0.48[c]
5 to 7 times per week	165 (19.9)	47 (15.6)	9 (13.2)	106 (20.0)	59 (19.7)		
1 to 4 times per week	47 (5.7)	12 (4.0)	2 (2.9)	30 (5.7)	17 (5.7)		
Less than once a week	18 (2.2)	9 (3.0)	2 (2.9)	15 (2.8)	3 (1.0)		
Does not use	595 (71.7)	229 (76.1)	55 (80.9)	375 (70.8)	220 (73.3)		
Iron						0.79[c]	0.65[c]
5 to 7 times per week	121 (14.8)	44 (14.8)	7 (10.4)	83 (15.8)	38 (13.1)		
1 to 4 times per week	44 (5.4)	15 (5.0)	3 (4.5)	31 (5.9)	13 (4.5)		
Less than once a week	13 (1.6)	6 (2.0)	2 (3.0)	9 (1.7)	4 (1.4)		
Does not use	633 (77.7)	229 (76.8)	55 (82.1)	400 (76.2)	233 (80.3)		
Fish oil						0.53[c]	0.38[c]
5 to 7 times per week	219 (25.6)	77 (24.7)	14 (19.7)	153 (27.4)	66 (22.1)		
1 to 4 times per week	48 (5.6)	16 (5.1)	7 (9.9)	29 (5.2)	19 (6.4)		
Less than once a week	22 (2.6)	6 (1.9)	1 (1.4)	16 (2.9)	6 (2.0)		
Does not use	565 (65.9)	211 (67.6)	49 (69.0)	358 (64.2)	207 (69.2)		
Probiotics						0.40[c]	0.015[c]
5 to 7 times per week	164 (19.6)	54 (17.9)	14 (19.4)	115 (21.1)	49 (16.9)		
1 to 4 times per week	49 (5.9)	22 (7.3)	3 (4.2)	40 (7.3)	9 (3.1)		
Less than once a week	53 (6.3)	25 (8.3)	2 (2.8)	38 (7.0)	15 (5.2)		
Does not use	566 (67.8)	199 (65.9)	53 (73.6)	350 (64.2)	216 (74.5)		

GDM, gestational diabetes. [a]between history of GDM groups [b]between normal-weight group and group with overweight/obesity [c]Fisher's exact test.

Table S3. Dietary quality and physical activity in the women subdivided by prepregnancy body mass index (BMI) and history of gestational diabetes, primiparous women excluded.

	**Women with normal weight, no history of GDM**	**Women with over-weight/ obesity, no history of GDM**	**Women with normal weight, history of GDM**	**Women with over-weight/ obesity, history of GDM**	
	n = 252	*n* = 131	*n* = 31	*n* = 55	*p*-value
IDQ score, mean (SD)	9.7 (2.1)	8.7 (2.2)	9.7 (2.2)	9.0 (2.3)	<0.001[a]
Good dietary quality, *n* (%)	131 (52.4)	42 (32.1)	16 (55.2)	22 (40.7)	0.001[b]
MET-index (MET h/wk), median (IQR)	7.5 (10.0)	4.8 (10.9)	7.5 (17.6)	4.8 (5.6)	0.079[c]
Categorized MET-index, *n* (%)					0.21[d]
Light LTPA	120 (48.0)	78 (60.0)	13 (43.3)	31 (56.4)	
Moderate LTPA	104 (41.6)	42 (32.3)	13 (43.3)	22 (40.0)	
Vigorous LTPA	26 (10.4)	10 (7.7)	4 (13.3)	2 (3.6)	

IDQ, Index of Diet Quality; SD, standard deviation; MET, metabolic equivalent; IQR, interquartile range, LTPA, leisure-time physical activity. [a]One-way ANOVA [b]Chi-square test [c]Kruskal-Wallis test [d]Fisher's exact test.
